# A Case of Intermittent Left Bundle Branch Block

**DOI:** 10.7759/cureus.15851

**Published:** 2021-06-23

**Authors:** Syed M Saad, Faran S Polani, Paul LeLorier

**Affiliations:** 1 Cardiology, Louisiana State University Health Sciences Center, New Orleans, USA; 2 Hematology and Oncology, Washington University School of Medicine, Saint Louis, USA

**Keywords:** intermittent left bundle branch block, ekg

## Abstract

An 82-year-old woman with uncontrolled hypertension and occasional exertional dyspnea was found to be in intermittent left bundle branch block (LBBB). Her laboratory results, echocardiogram, and ischemic workup were unremarkable. This case highlights that intermittent LBBB is not always associated with coronary ischemia, vasospasm, blunt cardiac injury, drugs, and high catecholaminergic or inflammatory states.

## Introduction

Bundle branch blocks and hemiblocks are usually stable and remain unchanged irrespective of the cardiac cycle length. Left bundle branch block (LBBB) usually results from conduction system degeneration or myocardial pathology [1]. Patients with LBBB have altered pattern of left ventricular activation and contraction, causing changes in left ventricular mechanics, perfusion, and workload, leading to pathologic cardiac remodeling and heart failure over time [2]. Though asymptomatic LBBB is uncommon, it carries with it a slightly increased mortality [3]. Not infrequently, LBBB appearance depends on the duration of the cardiac cycle, so it manifests and disappears with changes in heart rate [2]. This may not represent a pathological phenomenon, since changes in cardiac cycle can also result in aberrant conduction. 

Intermittent LBBB is an uncommon conduction disturbance with very few cases reported in the literature. It was first described by Vessel in 1949 as unstable bundle branch block [4]. In intermittent LBBB, normal QRS alternates with bundle branch block pattern QRS. This 2:1 LBBB pattern is mostly associated with tachycardia, aortic stenosis, ischemic heart disease, blunt cardiac injury, dilated cardiomyopathy, infective endocarditis, digoxin toxicity, and anesthesia induction [5,6]. The management and prognosis of intermittent LBBB depend on the etiology along with the presence or absence of organic heart disease. Patients often develop symptoms and persistent LBBB [7]. Focused history with home medications, labs, echocardiogram, and ischemic workup are of paramount importance. We report an interesting case of intermittent LBBB with otherwise unremarkable diagnostic workup. 

## Case presentation

**Figure 1:**
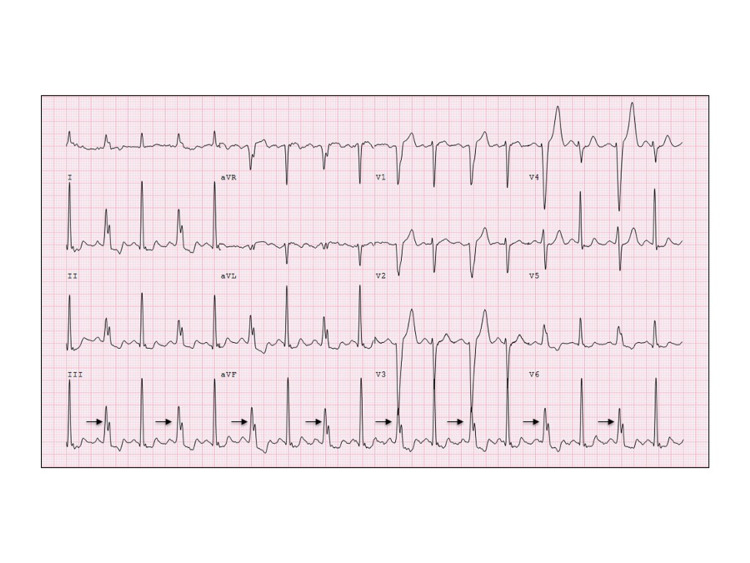
EKG Interpretation: Sinus rhythm with intermittent LBBB at 98 beats/min. The arrows indicate alternating beats with LBBB. LBBB, left bundle branch block.

An 82-year-old Caucasian female was referred to cardiology clinic for an abnormal EKG. She reported rare episodes of dyspnea on exertion but denied chest pain, palpitations, lightheadedness, syncope, and fatigue. EKG showed intermittent LBBB (Figure 1). Her blood pressure was 182/86 mmHg, pulse 92 bpm, respiratory rate 14, and oxygen saturation by pulse oximeter was 99% on room air. On physical examination, she appeared appropriate for age and was not in any distress. Chest auscultation revealed normal S1 and S2 with no heart murmurs and normal vesicular breathing. Jugular veins were not distended. There was no peripheral edema, cyanosis, or clubbing. Pules were intact and symmetric in all four extremities. She did not have any comorbidities and was not on any prescribed medicines. Blood workup including complete blood count, comprehensive metabolic panel, brain natriuretic peptide, and thyroid-stimulating hormone was within normal range. Chest radiogram did not show any acute cardiopulmonary abnormality, and cardiac silhouette was within normal limits. 

Echocardiogram demonstrated a left ventricular ejection fraction of 70%. All chamber sizes were within normal range with normal diastolic and valvular functions. Interventricular septal motion on echocardiogram was also unremarkable as she was in sinus rhythm with narrow QRS complexes. Holter monitor (24 hours) showed sinus rhythm with narrow QRS complexes alternating with intermittent LBBB at faster heart rates. Patient remained asymptomatic during monitoring. Myocardial perfusion single-photon emission computed tomography (SPECT) showed normal and homogeneous perfusion of all left ventricular wall segments at rest as well as with stress. On the quantitative gated SPECT study, there was normal myocardial thickening and contractility of all ventricular wall segments. Calculated ejection fraction was 70%. She was started on lisinopril 10 mg along with lifestyle modification for optimal blood pressure control. She continued to do well as she was followed closely in clinic for three years. Subsequent routine EKGs had either normal conduction or intermittent LBBB pattern with faster heart rates. 

Patient's electrocardiogram revealed a normal QRS complex alternating with an LBBB in a 2:1 pattern due to intermittent delay in the left bundle branch with constant PR, PP, and RR intervals. LBBB typically has a QRS duration of greater than 120 ms, and in comparison to ventricular ectopy, it has dominant S wave in V1, broad monophasic R wave in leads I and V6, and prolonged R wave peak time of greater than 60 ms in lead V6.

## Discussion

Intermittent LBBB is a rare occurrence. In literature, it has been reported with exercise, tachycardia, ischemic and hypertensive heart disease, hyperkalemia, drugs, anesthesia, pulmonary embolism, chest trauma, and cardiac interventional procedures [5,6]. Patients with intermittent LBBB may develop persistent LBBB; therefore, it is imperative to rule out reversible conditions and to follow them closely. Occasionally patients may present with acute left ventricular systolic and/or diastolic dysfunction with other conduction disturbances leading to lightheadedness and syncope. Exclusion of ischemia in the setting of episodic LBBB is of great importance. Our patient's electrolytes were within the normal range. She did not have structural heart disease and ischemia was ruled out as echocardiogram and myocardial SPECT were essentially unremarkable.

**Figure 2:**
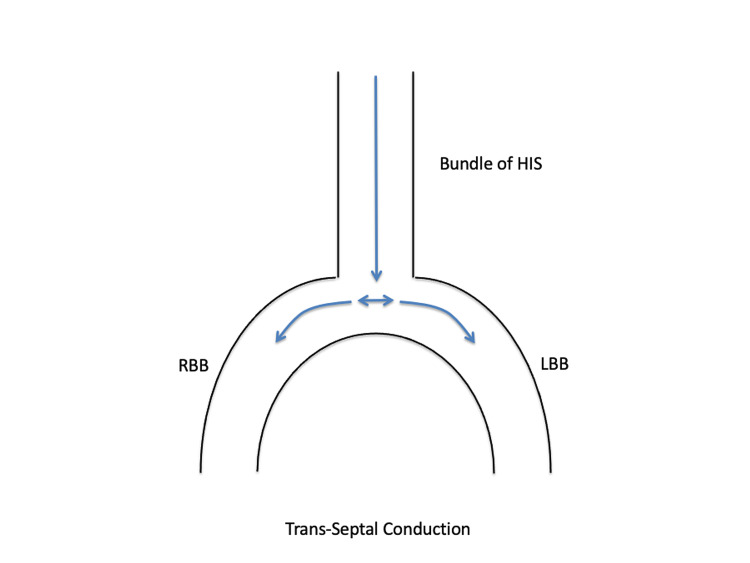
Normal Conduction Activation of the ventricles takes place by impulses entering via the bundle of His; they  travel to the LBB and RBB into the intricate system of Purkinje fibers depolarizing both ventricles. RBB, right bundle branch; LBB, left bundle branch.

**Figure 3:**
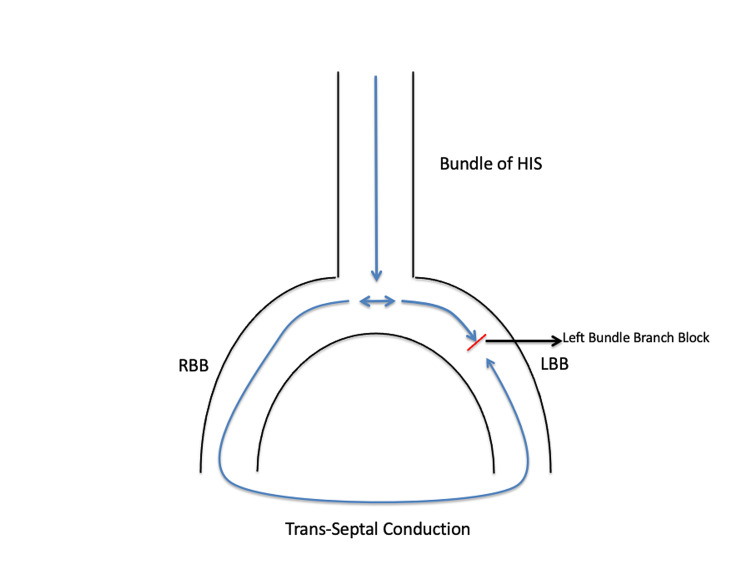
LBBB (1:1 LBBB pattern) The left conduction system of the heart is depolarized retrogradely from the RBB through the septum from right to left. RBB, right bundle branch; LBB, left bundle branch; LBBB, left bundle branch block.

Normally the activation of the left ventricle takes place by impulses entering the ventricle via the bundle of His; they then travel to the left and right bundle branches in the septum and from there into the intricate system of Purkinje fibers depolarizing both the ventricles (Figure 2) [8]. When the left bundle branch is injured, or when it is still in its effective refractory period (ERP) and the sinus cycle length is shorter than the left bundle ERP, it gets activated retrogradely from the right bundle branch via the septum called concealed trans-septal conduction [9]. This results in a continuous 1:1 LBBB pattern on the EKG (Figure 3). Consequently, the total time for left ventricular depolarization is prolonged and the QRS complex is abnormally wide.

**Figure 4:**
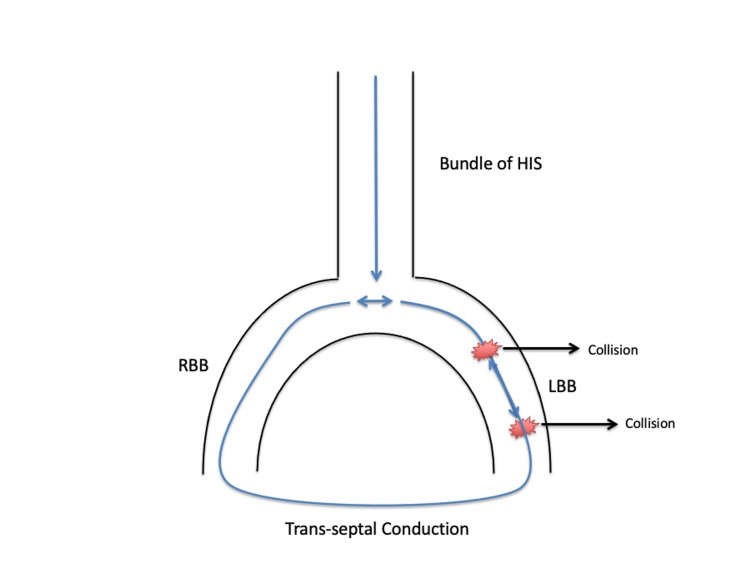
Phase 3 Block During tachycardia, the sinus impulse that is already blocked anterogradely in the LBB also fails to depolarize LBB retrogradely due to frequency (tachycardia)-dependent block. The following impulse finds the LBB in a non-refractory state and is conducted normally resulting in a 2:1 LBBB pattern on EKG. RBB, right bundle branch; LBB, left bundle branch; LBBB, left bundle branch block.

There are two proposed mechanisms of intermittent LBBB: tachycardia-dependent bidirectional block and supernormal conduction [10]. In tachycardia-dependent bidirectional block, the sinus impulse that is already blocked anterogradely in the LBB also fails to depolarize LBB retrogradely due to frequency (tachycardia)-dependent block. This is called phase 3 block and is shown in Figure 4 resulting in an LBBB pattern on EKG [10]. The next sinus beat will find the LBB in a non-refractory phase and conducts normally (2:1 LBBB pattern), since LBB was not depolarized at all (neither anterograde nor retrograde) during the bidirectional block. 

Supernormal conduction results in a 2:1 LBBB pattern when a beat is conducted better than expected in patients with LBBB pattern [10]. In this scenario, there is supernormal excitability in the late phase of cardiac tissue repolarization. The incoming impulse conducts in the supernormal phase of the LBB with narrow QRS complexes after which the LBB becomes refractory again resulting in a 2:1 LBBB pattern.

In patients with intermittent LBBB, it is not uncommon to observe periods of sinus rhythm with alternating (2:1) bundle branch block pattern at faster heart rates as in our patient, and they essentially may not have any underlying pathological phenomenon. Till her last visit she had been doing well without progression of her intermittent LBBB to complete bundle branch block. 

## Conclusions

Owing to our patient’s rare symptom of dyspnea on exertion and the association of her intermittent LBBB commonly with coronary artery disease, echocardiogram and myocardial perfusion imaging were ordered. Both the studies were unremarkable. EKGs on subsequent visits did not progress to complete LBBB. With optimal management of blood pressure, her dyspnea resolved. 
